# Daytime napping and successful aging among older adults in China: a cross-sectional study

**DOI:** 10.1186/s12877-019-1408-4

**Published:** 2020-01-02

**Authors:** Chunyu Xin, Baiyang Zhang, Shu Fang, Junmin Zhou

**Affiliations:** 1 West China School of Public Health and West China Fourth Hospital, Sichuan University, Chengdu, 610041 Sichuan China; 2 School of Public Health, Li Ka Shing Faculty of Medicine, The University of Hong Kong, 21 Sassoon Road, Hong Kong, 999077 Pokfulam China

**Keywords:** Chinese health and retirement longitudinal study, Older adults, Daytime napping, Successful aging

## Abstract

**Background:**

The study aimed to examine the association between daytime napping and successful aging (including its five dimensions, “low probability of disease,” “no disease-related disability,” “high cognitive functioning,” “high physical functioning,” and “active engagement with life”) among China’s older adults using data from the Chinese Health and Retirement Longitudinal Study conducted in 2015.

**Methods:**

Cross-sectional data were used in the analysis. Multivariable logistic regressions were conducted to investigate the association between daytime napping and successful aging, and stratified analyses were performed to explore differences in nighttime sleep duration.

**Results:**

A total of 7469 participants were included in the analysis. Daytime napping was prevalent in China’s older adults (59.3%). The proportion of study participants with “successful aging” was 13.7%. Additionally, 48.6, 91.7, 54.1, 78.5, and 49.1% participants achieved “low probability of disease,” “no disease-related disability,” “high cognitive functioning,” “high physical functioning,” and “active engagement with life,” respectively. Compared with the 0 min/day napping group, the > 60 min/day napping group was associated with a lower probability of achieving successful aging (OR, 0.762; 95% CI, 0.583–0.996). In the nighttime sleep duration stratification, the findings showed that in the ≥8 h/night group, napping > 60 min per day was associated with a lower likelihood of aging successfully (OR, 0.617; 95% CI, 0.387–0.984). Considering the five dimensions of successful aging, moderate and long daytime napping were negatively associated with “low probability of disease”; long daytime napping had negative associations with “no disease-related disability” and “high physical functioning”; moderate daytime napping had positive associations with “high cognitive functioning” and “active engagement with life.”

**Conclusions:**

Long daytime napping showed a lower likelihood of successful aging among the elderly in China. Special attention is necessary for elderly people who sleep for longer duration both during day and night. Biological and social factors affecting the relationship between daytime napping and successful aging need to be explored in depth in the future.

## Background

The global population aged 60 years or over has expanded rapidly in recent years, reaching 901 million in 2015 [1]. By 2050, it is projected to reach 2.1 billion, accounting for at least 20% of the total population [1]. In China, it also remains a daunting health challenge. According to the sixth Chinese national population census in 2010 [2], the proportion of elderly individuals aged 60 years or over reached 13.3% and it is projected to increase to 19.3% by 2025. Aging is associated with a decline in cognitive functioning and social relationships [3], physical impairment [4], and a majority of chronic diseases [5], and, thus, results in poor quality of life and strong dependency on others. Moreover, life expectancy at birth had been projected to rise from 69 years in 1990 to 75 years in 2013 [6]. Living longer may lead to more age-related problems.

Successful aging, which is regarded as a “gold standard” for assessing the health conditions of older people [7], implies aging in adults associated with no diseases, high physical and cognitive functioning, and active social engagement [8, 9]. The prevalence of successful aging among older adults reported by previous studies in several countries ranged from 11.9 to 64.6% [10–12]. In China, the proportion is relatively low, reaching only 13.3% [13]. The low prevalence of successful aging warrants more attention in this country.

Daytime napping is a widely adopted behavior in many countries, and the prevalence of daytime napping increases with age [14]. In China, daytime napping is common among older adults. Studies found that 68.6% of the older Chinese exhibited habitual daytime napping [15], with an average nap duration of 52.6 mins [16]. Although daytime napping is popular in older adults, its overall effect on health remains unclear. Some studies revealed that daytime napping was associated with high cognitive functioning [17] and positive mood [18], while others indicated that it was associated with chronic diseases [19], depression [20], and physical impairment [21]. Therefore, it is necessary to thoroughly evaluate the association between daytime napping and health among older adults. Considering the aforementioned significance of successful aging in older adults and the comprehensive multidimensional constructs successful aging has covered, the primary objective of the study was to assess the association between daytime napping and successful aging among China’s older adults using a large nationally representative sample.

In addition, previous studies have shown significant association between nighttime sleep and successful aging [13] and have demonstrated combined effects of daytime napping and nighttime sleep on certain diseases [22, 23]. Thus, the secondary objective for the study was to examine differences in the association between daytime napping and successful aging in different nighttime sleep duration groups.

## Methods

### Study population

The Chinese Health and Retirement Longitudinal Study (CHARLS) aims to address the needs of scientific research related to Chinese aging population and to support scientific research on Chinese population aged 45 years and above. Four-stage, stratified, cluster sampling was performed to select participants. The study was conducted in 28 provinces and covered 150 counties/districts and 450 villages/urban communities across China. The baseline survey was conducted between June 2011 and March 2012. Participants are followed every 2 years, using a face-to-face computer-assisted personal interviews. Detailed information on the study can be accessed at http://charls.pku.edu.cn/index/en.html.

**Fig. 1:**
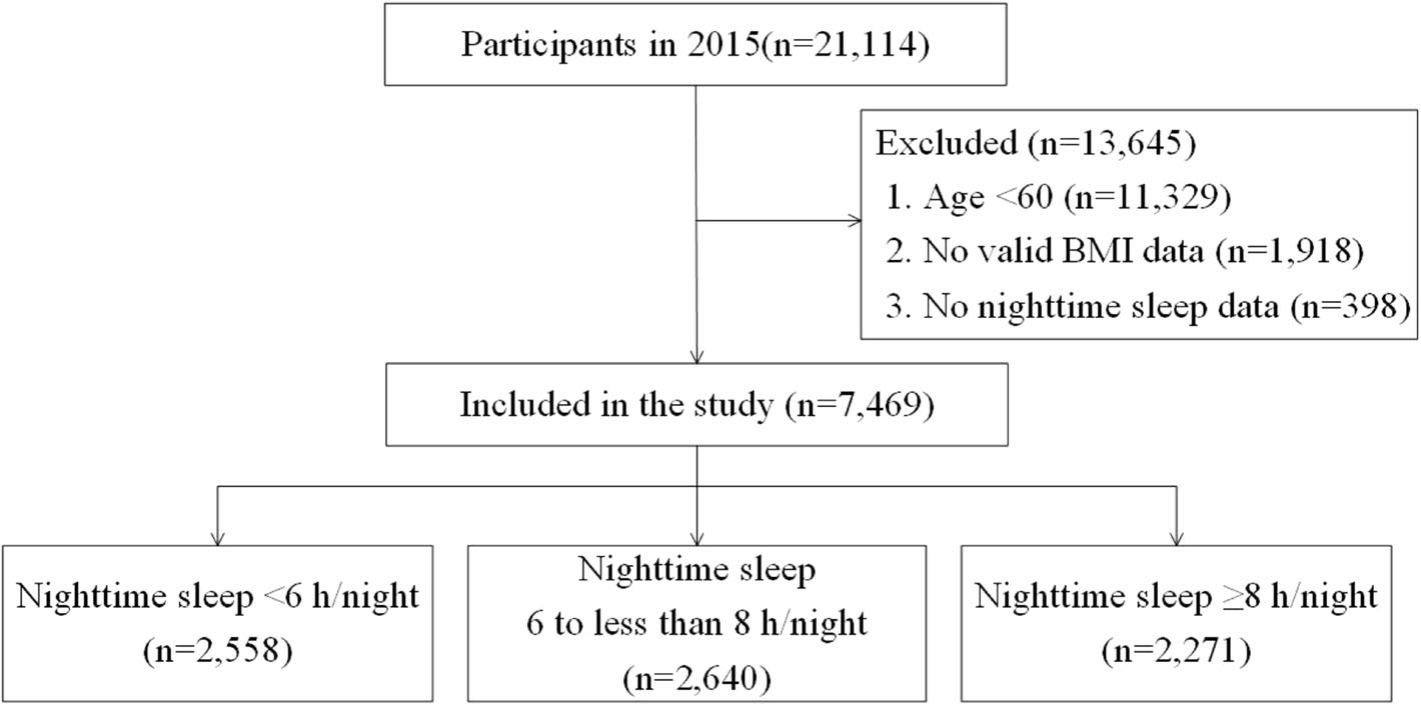
Sample included in the study

The data used in the study were obtained from the CHARLS conducted in 2015. As shown in Fig. 1, a total of 21,114 participants were investigated and 13,645 participants were excluded from the study for the following reasons:

(1) younger than 60 years (2); no inaccessible valid BMI data (3); no nighttime sleep data. The sample size in the final analysis was 7469. The sample sizes stratified by nighttime sleep duration were 2558, 2640, and 2271 in < 6 h/night, 6 to less than 8 h/night, and ≥ 8 h/night groups, respectively.

The secondary data used in the analysis are accessible to be downloaded publicly at http://charls.pku.edu.cn/pages/data/111/en.html. All the participants signed an informed consent before conducting interviews. Ethical approval for data collection was obtained from the Biomedical Ethics Review Committee of Peking University (IRB00001052–11015) [13].

### Measurements

#### Daytime napping

Duration of daytime napping was recorded by asking the participants “During the past month, how long (minutes) did you take a nap after lunch?” According to the self-reported duration of daytime napping, the participants were grouped into no daytime napping (0 min/day), moderate daytime napping (1–60 min/day) and long daytime napping (> 60 min/day) groups.

#### Successful aging

Based on Rowe and Kahn’s criteria of successful aging [8, 9], successful aging in the study was operationalized into five dimensions: [1] low probability of disease [2]; no disease-related disability [3]; high cognitive functioning [4]; high physical functioning [5]; active engagement with life. Participants who achieved all five dimensions were recorded as having achieved “successful aging.”

- Low probability of disease

Participants were classified as having “low probability of disease” if they did not report they had been diagnosed with any of the following major diseases (diabetes, cancer, chronic lung diseases, heart attack, stroke, emotional or psychiatric problems, and asthma) or risk factors of chronic diseases (hypertension and obesity) by doctors.

(b) No disease-related disability

“No disease-related disability” consists of performing six activities of daily living (ADLs) without difficulties, including dressing up, bathing or showering, eating, getting into or out of bed, using the toilet, controlling urination, and defecation.

(c) High cognitive functioning

As stated in previous studies [13, 24], respondents were defined to have “high cognitive functioning” if they accomplished the following tests with a median score or higher scores: stating the day of the year, month, day, week, and season; computing serial subtractions of 7 from 100 five times; and immediate and delayed recall of 10 words.

(d) High physical functioning

Three activities were used to assess the physical functioning: walking 100 m, getting up from a chair, and climbing several flights of stairs without rest. If they had no difficulties with these activities, they were regarded to have “high physical functioning.”

(e) Active engagement with life

Participants were classified as having “active engagement with life” if they provided history of interpersonal interactions and performing productive activities in the last month, such as “interacted with friends,” “played Ma-Jong, played chess, played cards, or went to the community club,” “provided help to family, friends, or neighbors who did not live with you,” “went to a sport, social, or other kind of club,” “took part in a community-related organization,” and other social activities.

#### Covariates

Covariates were grouped into three categories: socio-demographic variables, health behavior, and health status.

- Socio-demographic variables

Older adults were defined as people aged 60 years and above. Age was categorized into three groups (60–64, 65–74, and ≥ 75 years). Education level was divided into “no formal education (illiterate),” “did not finish primary school,” “sishu/homeschool/elementary school,” and “middle school and above.” Marital status was recorded as a dichotomous variable: cohabitated (married or not married), and others (married but not living with spouse temporarily for reasons such as work, separated, widowed, or never married). Community type was classified as “urban area” (including main city zone, transition zone between urban and rural areas, the town center, ZhenXiang area, and special area) and “rural area” (including township center and village) [25]. Annual household income, which was evaluated by aggregating income of the participants and their family members, was classified into four categories: less than 2000 Chinese Yuan Renminbi (CNY), 2001 CNY to 10,000 CNY, 10,001 CNY to 25,000 CNY, and more than 25,000 CNY (Yuan – US dollar exchange rate was about 6.2 Yuan per US dollar in 2015). The missing data of annual household income were derived by linear interpolation, an imputation technique.

(b) Health behavior

Tobacco use was recorded as “yes” (including current and former smokers) or “no.” Participants were classified into three categories based on alcohol consumption: “drank more than once a month in the past year,” “drank less than once a month in the past year,” and “no.”

Nighttime sleep duration was assessed by the question: “During the past month, how many hours of actual sleep did you get at night (average hours per night)? (This may be shorter than the number of hours you spend in bed.).” Responses were grouped into three tertiles—inadequate nighttime sleep (< 6 h/night), moderate nighttime sleep duration (6 to less than 8 h/night), and long nighttime sleep duration (≥8 h/night).

Low quality of nighttime sleep was estimated by appropriate response of respondents to the statement, “My sleep was restless” regarding the experience during the past week [13]. The responses were classified into four groups—rarely or never (< 1 day/week), sometimes or a few days (1–2 days/week), occasionally or a moderate number of days (3–4 days/week), and most or all days (5–7 days/week).

(c) Health status

Considering the differences in criteria of overweight and obesity in different countries, Body Mass Index (BMI) was defined according to the National Health and Family Planning Commission of the People’s Republic of China; underweight was defined as BMI < 18.50 kg/m^2^, normal weight as BMI of 18.50–23.99 kg/m^2^, overweight as BMI of 24.00–27.99 kg/m^2^ and obesity as BMI ≥28.00 kg/m^2^ [26].

### Statistical analysis

Frequencies and percentages were evaluated to describe the samples. Multivariable logistic regressions were conducted to examine the association of daytime napping with successful aging and its five dimensions after adjusting for possible confounders (sex, age, education level, marital status, community type, annual household income, tobacco use, alcohol consumption, physical activity, self-reported nighttime sleep duration, and low quality of nighttime sleep). Furthermore, stratified analyses were used for in-depth exploration of differences in the association of daytime napping with successful aging and its five dimensions among the three nighttime sleep duration groups. The odds ratios (ORs) and corresponding 95% confidence intervals (95% CIs) were assessed. The association was statistically significant if the 2-sided *p*-value was < 0.05. SPSS version 20.0 was used to perform all statistical analyses [27].

## Results

Table 1 showed the characteristics of participants with different nighttime sleep durations. Overall, the proportion of males was 50.0% among all participants (*n* = 3734). The mean age of the participants was 67.95 years and 1227 participants (16.4%) were aged 75 years or above.

**Table 1:** Characteristics of participants with different nighttime duration

	TOTAL n (%)	Nighttime sleep duration (per night)		
		< 6 h n (%)	6 to less than 8 h n (%)	≥8 h n (%)
Successful aging				
No	6191 (86.1)	2156 (87.9)	2125 (83.0)	1910 (87.7)
Yes	999 (13.9)	296 (12.1)	435 (17.0)	268 (12.3)
Low probability of disease				
No	3822 (51.2)	1363 (53.3)	1359 (51.5)	1100 (48.4)
Yes	3647 (48.8)	1195 (46.7)	1281 (48.5)	1171 (51.6)
No disease-related disability				
No	566 (7.6)	262 (10.3)	159 (6.0)	145 (6.4)
Yes	6884 (92.4)	2289 (89.7)	2475 (94.0)	2120 (93.6)
High cognitive functioning				
No	3303 (45.2)	1253 (50.2)	940 (36.2)	1110 (50.2)
Yes	4000 (54.8)	1243 (49.8)	1657 (63.8)	1100 (49.8)
High physical functioning				
No	1480 (20.1)	652 (25.9)	413 (15.9)	415 (18.5)
Yes	5883 (79.9)	1866 (74.1)	2190 (84.1)	1827 (81.5)
Active engagement with life				
No	3660 (49.0)	1277 (49.9)	1198 (45.4)	1185 (52.2)
Yes	3809 (51.0)	1281 (50.1)	1442 (54.6)	1086 (47.8)
Daytime napping (per day)				
0 min	3012 (40.5)	1220 (47.9)	968 (36.8)	824 (36.6)
1–60 min	2928 (39.4)	926 (36.4)	1149 (43.6)	853 (37.9)
> 60 min	1493 (20.1)	401 (15.7)	517 (19.6)	575 (25.5)
Sex				
Male	3734 (50.0)	1080 (42.2)	1436 (54.4)	1218 (53.6)
Female	3735 (50.0)	1478 (57.8)	1204 (45.6)	1053 (46.4)
Age				
60–64	2791 (37.4)	870 (34.0)	1093 (41.4)	828 (36.5)
65–74	3451 (46.2)	1212 (47.4)	1211 (45.9)	1028 (45.3)
≥ 75	1227 (16.4)	476 (18.6)	336 (12.7)	415 (18.3)
Community type				
Rural	5588 (76.0)	1940 (77.1)	1819 (70.3)	1829 (81.5)
Urban	1761 (24.0)	577 (22.9)	770 (29.7)	414 (18.5)
Education				
No formal education	2428 (33.3)	940 (37.5)	702 (27.4)	786 (35.5)
Did not finish primary school	1574 (21.6)	541 (21.6)	545 (21.2)	488 (22.1)
Sishu/Homeschool/Elementary school	1745 (24.0)	553 (22.1)	658 (25.6)	534 (24.2)
Middle school and above	1535 (21.1)	471 (18.8)	661 (25.8)	403 (18.2)
Marital status				
Cohabited (married or not)	5823 (78.0)	1880 (73.5)	2159 (81.8)	1784 (78.6)
Did not cohabit (Separated/divorced/widowed and never married)	1646 (22.0)	678 (26.5)	481 (18.2)	487 (21.4)
Annual household income				
≤ 2000 CNY	2474 (33.1)	917 (35.8)	777 (29.4)	780 (34.3)
2001–10,000 CNY	1796 (24.1)	657 (25.7)	591 (22.4)	548 (24.1)
10,001–25,000 CNY	1485 (19.9)	483 (18.9)	565 (21.4)	437 (19.2)
> 25,000 CNY	1714 (22.9)	501 (19.6)	707 (26.8)	506 (22.3)
Tobacco use				
No	3942 (52.8)	1466 (57.4)	1325 (50.2)	1151 (50.7)
Yes	3523 (47.2)	1090 (42.6)	1314 (49.8)	1119 (49.3)
Alcohol consumption				
No	5001 (67.0)	1759 (68.8)	1705 (64.6)	1537 (67.7)
< once a month	550 (7.4)	187 (7.3)	218 (8.3)	145 (6.4)
≥ once a month	1915 (25.6)	611 (23.9)	716 (27.1)	588 (25.9)
Physical activity				
No	4280 (57.3)	1482 (57.9)	1487 (56.3)	1311 (57.7)
Yes	3189 (42.7)	1076 (42.1)	1153 (43.7)	960 (42.3)
Low quality of nighttime sleep				
< 1 day/week	3805 (51.3)	716 (28.1)	1511 (57.6)	1578 (70.1)
1–2 days/week	1008 (13.6)	311 (12.2)	421 (16.1)	276 (12.3)
3–4 days/week	978 (13.2)	436 (17.1)	350 (13.3)	192 (8.5)
5–7 days/week	1626 (21.9)	1082 (42.5)	340 (13.0)	204 (9.1)
Body Mass Index				
Normal	3857 (51.6)	1357 (53.0)	1333 (50.5)	1167 (51.4)
Underweight	569 (7.7)	209 (8.2)	177 (6.7)	183 (8.1)
Overweight	2249 (30.1)	728 (28.5)	832 (31.5)	689 (30.3)
Obesity	794 (10.6)	264 (10.3)	298 (11.3)	232 (10.2)
Nighttime sleep duration (per night)				
< 6 h	2558 (34.3)	–	–	–
6 to less than 8 h	2640 (35.3)	–	–	–
≥ 8 h	2271 (30.4)	–	–	–

Among a total of 7469 respondents, 40.5% reported no daytime napping (0 min/day), 39.4% reported moderate daytime napping (1–60 min/day) and 20.1% reported long daytime napping (> 60 mins/day). The mean duration of daytime napping was 40.8 ± 0.5 mins in all respondents and 68.6 ± 0.6 mins in participants who habitually napped.

The proportion of participants with “successful aging” was 13.9%. Additionally, among the total participants, 48.8, 92.4, 54.8, 79.9, and 51.0% achieved “low probability of disease,” “no disease-related disability,” “high cognitive functioning,” “high physical functioning” and “active engagement with life,” respectively.

Table 2 shows the association between daytime napping and successful aging before and after stratification by nighttime sleep duration after adjusting for possible confounders. Compared with the 0 min/day napping group, the > 60 mins/day napping group was found to be associated with lower odds of achieving successful aging (OR, 0.762; 95% CI, 0.583–0.996). Participants who did not achieve successful aging were more likely to be older in age, less educated, living in rural areas, having a low-income, performing inadequate physical activities and reporting low quality of nighttime sleep. In the subgroup analyses, stratified by nighttime sleep duration, napping more than 60 mins/day was associated with a lower likelihood of achieving successful aging in the long nighttime sleep duration group (≥8 h/night) (OR, 0.617; 95% CI, 0.387–0.984).

**Table 2:** Multivariable Logistic Regression on Daytime Napping and Successful Aging

	TOTAL OR (95% CI)	Nighttime sleep duration (per night)		
		< 6 h OR (95% CI)	6 to less than 8 h OR (95% CI)	≥8 h OR (95% CI)
Daytime napping (per day)				
0 min (ref)	–	–	–	–
1–60 min	1.012 (0.829–1.234)	1. 270 (0.887–1.817)	1.004 (0.736–1.370)	0.818 (0.559–1.196)
> 60 min	**0.762 (0.583–0.996)**	0.875 (0.516–1.484)	0.837 (0.550–1.276)	**0.617 (0.387–0.984)**
Sex				
Male (ref)	–	–	–	–
Female	1.060 (0.804–1.396)	0.727 (0.433–1.222)	1.083 (0.706–1.662)	1.541 (0.922–2.573)
Age				
60–64(ref)	–	–	–	–
65–74	**0.614 (0.507–0.745)**	**0.639 (0.447–0.914)**	**0.597 (0.443–0.804)**	**0.620 (0.429–0.897)**
≥ 75	**0.451 (0.327–0.621)**	**0.344 (0.189–0.625)**	**0.468 (0.274–0.798)**	**0.559 (0.320–0.978)**
Education				
No formal education (illiterate) (ref)	–	–	–	–
Did not finish primary school	**2.170 (1.606–2.933)**	**1.906 (1.092–3.326)**	**1.947 (1.192–3.180)**	**2.635 (1.541–4.504)**
Sishu/homeschool/elementary school	**3.053 (2.280–4.087)**	**2.937 (1.717–5.022)**	**2.875 (1.801–4.589)**	**3.439 (2.018–5.861)**
Middle school and above	**4.952 (3.685–6.656)**	**5.261 (3.098–8.934)**	**4.252 (2.640–6.849)**	**5.887 (3.394–10.209)**
Marital status				
Married and cohabited (ref)	–	–	–	–
Other (Separated/divorced/widowed and never married)	0.783 (0.612–1.003)	0.995 (0.663–1.494)	0.757 (0.501–1.144)	0.590 (0.358–0.972)
Community type				
Rural area (ref)	–	–	–	–
Urban area	**1.271 (1.030–1.568)**	**1.591 (1.088–2.328)**	1.070 (0.774–1.480)	1.391 (0.923–2.094)
Annual household income				
≤ 2000 CNY (ref)	–	–	–	–
2000 < x ≤ 10,000 CNY	1.232 (0.953–1.593)	1.042 (0.667–1.629)	1.278 (0.839–1.947)	1.447 (0.892–2.348)
10,000 < x ≤ 25,000 CNY	1.177 (0.901–1.537)	0.932 (0.572–1.518)	1.268 (0.832–1.932)	1.340 (0.803–2.236)
> 25,000 CNY	**1. 456 (1.135–1.868)**	1.039 (0.649–1.662)	1.429 (0.960–2.127)	**2.010 (1.274–3.170)**
Tobacco use				
No (ref)	–	–	–	–
Yes	1.078 (0.833–1.395)	0.845 (0.515–1.384)	1.220 (0.817–1.822)	1.157 (0.721–1.856)
Alcohol consumption				
No (ref)	–	–	–	–
< once a month	**1.350 (1.033–1.764)**	**1.819 (1.123–2.948)**	1.033 (0.679–1.571)	1.559 (0.933–2.606)
≥ once a month	0.930 (0.710–1.217)	0.911 (0.543–1.529)	0.785 (0.516–1.194)	1.224 (0.748–2.004)
Physical activity				
No (ref)	–	–	–	–
Yes	**1.353 (1.130–1.620)**	1.185 (0.847–1.658)	**1.351 (1.019–1.790)**	**1.545 (1.100–2.169)**
Low quality of nighttime sleep				
< 1 day/week (ref)	–	–	–	–
1–2 days/week	1.078 (0.834–1.394)	1.272 (0.758–2.134)	0.843 (0.570–1.248)	1.366 (0.854–2.187)
3–4 days/week	**0.615 (0.452–0.837)**	0.735 (0.433–1.248)	**0.594 (0.374–0.943)**	0.522 (0.245–1.111)
5–7 days/week	**0.659 (0.501–0.867)**	0.802 (0.531–1.212)	**0.468 (0.277–0.791)**	0.843 (0.429–1.656)
Nighttime sleep duration (per night)				
6 to less than 8 h (ref)	–	–	–	–
< 6 h	0.926 (0.734–1.169)	–	–	–
≥ 8 h	0.936 (0.750–1.168)	–	–	–

Bold values indicate statistical significance

The association between daytime napping and the five dimensions of successful aging after adjustment for possible confounders are shown in Table 3.

**Table 3:** Association between daytime napping and five components of successful aging among the total population and stratified by the nighttime sleep duration groups

	Low probability of disease			
	TOTAL OR (95%CI)	Nighttime sleep duration (per night)		
		< 6 h OR (95%CI)	6 to less than 8 h OR (95%CI)	≥8 h OR (95%CI)
Daytime napping (per day)				
0 min	1	1	1	1
1–60 min	**0.776 (0.686–0.878)**	0.818 (0.666–1.004)	**0.740 (0.599–0.916)**	**0.753 (0.598–0.949)**
> 60 min	**0.761 (0.651–0.889)**	0.770 (0.581–1.020)	**0.720 (0.548–0.947)**	**0.759 (0.582–0.988)**
	No disease-related disability			
	TOTAL OR (95%CI)	Nighttime sleep duration (per night)		
		< 6 h OR (95%CI)	6 to less than 8 h OR (95%CI)	≥8 h OR (95%CI)
Daytime napping (per day)				
0 min	1	1	1	1
1–60 min	0.848 (0.674–1.067)	0.931 (0.675–1.285)	0.746 (0.462–1.203)	0.730 (0.448–1.188)
> 60 min	**0.595 (0.455–0.777)**	0.859 (0.558–1.324)	**0.355 (0.213–0.591)**	**0.581 (0.345–0.980)**
	High cognitive functioning			
	TOTAL OR (95%CI)	Nighttime sleep duration (per night)		
		< 6 h OR (95%CI)	6 to less than 8 h OR (95%CI)	≥8 h OR (95%CI)
Daytime napping (per day)				
0 min	1	1	1	1
1–60 min	**1.233 (1.063–1.430)**	**1.608 (1.257–2.056)**	1.187 (0.918–1.535)	0.925 (0.699–1.223)
> 60 min	1.062 (0.883–1.277)	1.226 (0.880–1.707)	0.875 (0.634–1.209)	1.060 (0.774–1.452)
	High physical functioning			
	TOTAL OR (95%CI)	Nighttime sleep duration (per night)		
		< 6 h OR (95%CI)	6 to less than 8 h OR (95%CI)	≥8 h OR (95%CI)
Daytime napping (per day)				
0 min	1	1	1	1
1–60 min	0.927 (0.794–1.083)	1.023 (0.808–1.295)	0.783 (0.585–1.049)	0.952 (0.704–1.287)
> 60 min	**0.775 (0.640–0.937)**	0.870 (0.632–1.196)	**0.573 (0.405–0.811)**	0.881 (0.627–1.238)
	Active engagement with life			
	TOTAL OR (95%CI)	Nighttime sleep duration (per night)		
		< 6 h OR (95%CI)	6 to less than 8 h OR (95%CI)	≥8 h OR (95%CI)
Daytime napping (per day)				
0 min	1	1	1	1
1–60 min	**1.183 (1.044–1.339)**	**1.246 (1.014–1.530)**	1.091 (0.880–1.353)	1.200 (0.949–1.519)
> 60 min	1.120 (0.958–1.310)	1.223 (0.923–1.619)	0.880 (0.667–1.159)	1.300(0.993–1.700)

^a^Bold values indicate statistical significance

^b^BMI was not adjusted in the “low probability of disease” since obesity was regarded as a risk factor that was included in the dependent variable

Compared with 0 min/day napping group, participants who reported a moderate duration of napping (1–60 min/day) or long duration of napping (> 60 mins/day) had lower odds of “low probability of disease” (OR, 0.776; 95% CI, 0.686–0.878 and OR, 0.761; 95% CI, 0.651–0.889, respectively). This association was significant in the 6 to less than 8 h/night group (OR, 0.740; 95% CI, 0.599–0.916) and the ≥8 h/night group (OR, 0.753; 95% CI, 0.598–0.949), while it was not significant in the < 6 h/night group in subgroup analysis.

In comparison with the 0 min/day napping group, the > 60 min/day napping group was less likely to achieve “no disease-related disability” (OR, 0.595; 95% CI, 0.455–0.777). However, the association was only significant in the 6 to less than 8 h/night group (OR, 0.355; 95% CI, 0.213–0.591) and the ≥8 h/night group (OR, 0.581; 95% CI, 0.345–0.980) in stratified analysis.

Compared with the 0 min/day napping group, those napped 1–60 min/day were more likely to achieve “high cognitive functioning” (OR, 1.233; 95% CI, 1.063–1.430). After stratification, the association between daytime napping and “high cognitive functioning” was only found to be significant in the < 6 h/night group (OR, 1.608; 95% CI, 1.257–2.056).

In comparison with those who had no daytime napping (0 min/day), the > 60 min/day napping group had lower odds of having “high physical functioning” (OR, 0.775; 95% CI, 0.640–0.937). In stratified analysis, napping more than 60 mins/day was negatively associated with achieving “high physical functioning” in the 6 to less than 8 h/night group (OR, 0.573; 95% CI, 0.405–0.811) compared with the 0 min/day napping group.

The 1–60 min/day napping group was more likely to have “active engagement with life,” in comparison with the 0 min/day napping group (OR, 1.183; 95% CI, 1.044–1.339). After stratification, napping 1–60 min/day with < 6 h/night sleep was found to be significantly associated with respondents achieving “active engagement with life” (OR, 1.246; 95% CI, 1.014–1.530).

## Discussion

The study examined the association between daytime napping and successful aging among China’s older adults using multivariable logistic regressions and differences in the relationship based on nighttime sleep duration using stratified analysis. Findings suggest that compared with the 0 min/day napping group, the > 60 min/day napping group was found to be associated with lower odds of achieving successful aging, especially in the ≥8 h/night subgroup.

In our study, 59.3% of the older adults reported habitual daytime napping. Of the total participants, 39.2% reported moderate daytime napping duration and 20.1% reported long daytime napping duration. These findings add to current evidence supporting the fact that daytime napping is a widely adopted lifestyle behavior in older adults in China [16]. Of the total participants, 13.7% achieved “successful aging.” Regarding the five dimensions of successful aging, 48.8, 92.4, 54.8, 79.9 and 51.0% achieved “low probability of disease,” “no disease-related disability,” “high cognitive functioning,” “high physical functioning,” and “active engagement with life,” respectively. This was in accordance with a previous study conducted in 2013 [13], which reported that 13.3% of older adults in China achieved “successful aging,” and 41.9, 92.6, 54.5, 70.6, and 46.3% achieved “no major disease,” “no disability,” “high cognitive functioning,” “high physical functioning” and “active engagement with life,” respectively.

Our study showed that compared with the 0 min/day napping group, the > 60 min/day napping group was associated with a lower likelihood of successful aging. This was partially consistent with former studies, which demonstrated that long duration of daytime napping (> 60 min/day) was independently associated with major diseases [28], cognitive impairment [29], and physical activity deficit [30]. However, this association was only found to be significant in the ≥8 h/night group. Hence, respondents with long duration of both nighttime sleep (≥8 h/night) and long daytime napping (> 60 min/day) were less likely to achieve successful aging. As indicated by previous studies, both long duration of daytime napping and long duration of nighttime sleep were indicators of sleep disturbance [22] and both were associated with health issues independently [13, 31, 32]. Similar results were found regarding the association between daytime napping and stroke in a previous study, which reported that long daytime napping combined with long nighttime sleep duration was significantly associated with a high risk of stroke [23]. Therefore, our observation of differences in the association between daytime napping and successful aging based on duration of nighttime sleep could be partially corroborated by these findings.

The association between daytime napping and the five dimensions of successful aging varied. Daytime napping was positively associated with “high cognitive functioning” and “active engagement with life,” and was negatively related to “a low probability of disease,” “no disease-related disability,” and “high physical functioning.”

In comparison with the 0 min/day napping group, the napping groups (1–60 min/day, > 60 min/day) had lower odds of achieving “low probability of disease.” This was consistent with former studies that revealed that daytime napping was associated with respiratory [33], cardiovascular disease [34], stroke [23], cancer [35], depression [20], diabetes [36], asthma [37], hypertension [38], and obesity [39]. However, the association was only significant in the 6 to less than 8 h/night and ≥ 8 h/night groups, but not significant in the < 6 h/night group. A previous study demonstrated that long daytime napping and long nighttime sleep duration were jointly associated with diabetes [40]; this partially validated our findings.

Compared with the 0 min/day napping group, the > 60 min/day napping group had lower odds of having “no disease-related disability.” This could be partially explained by the findings in a previous study, which revealed that excessive daytime napping was associated with a higher limitation of performing instrumental activities of daily living (IADL) [21]. When stratified by nighttime sleep duration, the associations were only significant in the 6 to less than 8 h/night group and the ≥8 h/night group. This may be because difficulties with performing ADLs might result in impairment of mobility, and people with restricted mobility might have a tendency for excessive daytime napping and long duration of nighttime sleep to tackle the effects of sleep issues (e.g. sleep disturbance, sleep efficiency) [41].

Previous studies revealed that moderate duration of daytime napping was associated with improved waking cognitive performance [17] and alertness [42]. Although a different measure of cognitive functioning was used, our study found that moderate daytime napping (1–60 min/day) was associated with higher cognitive functioning. Moreover, this association was only found in the < 6 h/night group, which implies that participants with a combination of both moderate daytime napping and inadequate nighttime sleep were more likely to have “high cognitive functioning.” This may be explained by a former study [43], which showed that daytime napping played a compensatory role in the sleep routine.

Compared with those with no daytime napping (0 min/day), the > 60 min/day napping group had lower odds of “high physical functioning.” After stratification, the association was only found in the participants with 6 to less than 8 h/night sleep, but not in the participants with < 6 h/night or ≥ 8 h/night sleep. A previous study found that inadequate sleep and long nighttime sleep duration was associated with physical impairment and low neuromuscular performance [21]; this was partially supported by our observation that the proportion of participants with “high physical functioning” was comparatively low in the < 6 h/night (74.1%) and ≥ 8 h/night (81.5%) groups, so the effects of daytime napping on physical functioning in these two groups might have been diminished.

Compared with the participants who had 0 min/day napping, the participants in the 1–60 min/day napping group were more likely to have “active engagement with life.” In stratified analysis, this association was significant in those who had < 6 h/night sleep. This could be explained by our observation that the respondents who reported napping of 1–60 min/day were more likely to have a higher cognition in the < 6 h/night sleep group, considering that higher cognition increases the likelihood to actively engage with life [44].

There were some limitations to this study. First, there was no objective measurement in some variables (e.g. daytime napping, nighttime sleep duration and diagnosed diseases in successful aging), which might account for a possibility of recall bias. Second, in this cross-sectional study, the causal relationship between daytime napping and successful aging could not be established. Third, our study contains no qualitative data on daytime napping. Thus, one cannot disentangle the underlying mechanisms of the association between napping and successful aging. Fourth, some potential confounders (e.g. depression, medication use) of the relationship between daytime napping and successful aging were not incorporated in our study due to unavailability of data. Last, the study sample was conducted in China, where cultural norms and patterns of daytime napping may be different from that in other countries, thereby limiting the generalizability of our findings to other groups globally. Nevertheless, to our knowledge, this is the first study to examine the association between daytime napping and successful aging using a large nationally representative sample.

Future studies may benefit from using longitudinal data to explore the causal relationship and using objective measurements of sleep such as actigraphy and polysomnography [45] to reduce recall bias. Also, Mendelian randomization studies are suggested to be conducted to avoid residual and unmeasured confounding effects. Furthermore, biological and social factors affecting the relationship between daytime napping and successful aging need in-depth exploration in future research.

### Applications

Based on our study findings, interventions promoting successful aging could target older adults with long duration of daytime napping, especially those who sleep more than 8 h/night. Increased exercise and leisure activity opportunities could be provided for the elderly [46, 47] and psychosocial care could be encouraged to help older adults to attain successful aging [48].

For the older adults with cognitive impairment, moderate daytime napping could be recommended, especially among those whose nighttime sleep is inadequate. In addition, population with major diseases and risk factors for diseases (i.e. diabetes; cancer; chronic lung diseases, heart attack, stroke, emotional or psychiatric problems, asthma, hypertension, and obesity) may be recommended to avoid daytime napping, especially those who sleep 6 h and above per night. To improve the chances of successfully modifying and maintaining napping behavior, these recommendations could be provided by physicians in hospital settings [49] when older adults are diagnosed or followed up with health issues.

## Conclusion

The study primarily examined the association between daytime napping and successful aging among China’s older adults, and secondly it investigated the differences in the association in different nighttime sleep duration groups. Compared with the 0 min/day napping group, the > 60 min/day napping group was associated with lower odds of achieving successful aging, and significant association was only found in the ≥8 h/night group. Based on our findings, interventions promoting successful aging may be tailored to cater different populations. Biological and social factors affecting the relationship between daytime napping and successful aging need in-depth exploration in future.

## Data Availability

The data used in the study are accessible to be downloaded publicly at http://charls.pku.edu.cn/pages/data/111/en.html.

## Abbreviations

ADLs: Activities of daily living
BMI: Body Mass Index
CHARLS: Chinese Health and Retirement Longitudinal Study
CIs: Confidence intervals
CNY: Chinese Yuan Renminbi
IADL: Instrumental activities of daily living
ORs: Odds ratios
